# Supplementary material for: MetaRibo-Seq measures translation in microbiomes
Source: Nat Commun. 2020 Jun 29;11:3268. doi: 10.1038/s41467-020-17081-z (PMC7324362; doi:10.1038/s41467-020-17081-z)
Supplement: Supplementary file 10 — Supplementary Data 7 [file 41467_2020_17081_MOESM10_ESM.zip › File2/Confidence_High_Taxonomy/292412_out.krona.html]

Javascript must be enabled to view this page.

members
magnitude
magnitudeUnassigned
count
unassigned
taxon
rank

292412\_out

5

5
superkingdom
2

1
phylum
976

class
1
200643

171549
1
order

1
family
815

1
genus
816

species
1

SRS019787\_contig\_number\_8884
162156

1239
phylum
4

class
4
186801

186802
4
order

4
family
186803

genus
4
841


SRS013940\_contig\_number\_contig-100\_10538.103328SRS050925\_contig\_number\_30014SRS077552\_contig\_number\_18024SRS098061\_contig\_number\_11635
166486
species
4
